# The Impact of Ivy Leaf Dry Extract EA 575 on Subsequent Antibiotic Use and Its Therapeutic Value in Children and Adolescents with the Common Cold: A Retrospective Prescription Database Analysis

**DOI:** 10.3390/children12040518

**Published:** 2025-04-17

**Authors:** Christian Vogelberg, Georg Seifert, Simon Braun, Rebecca Zingel, Karel Kostev

**Affiliations:** 1 Paediatric Department, University Hospital Carl Gustav Carus Dresden, Technical University Dresden, 01069 Dresden, Germany; 2 Department of Paediatric Oncology/Haematology, Otto-Heubner Centre for Paediatric and Adolescent Medicine (OHC), Charité—Universitätsmedizin Berlin, 13353 Berlin, Germany; 3 Corporate Member of Freie Universität Berlin, Humboldt-Universität zu Berlin, Berlin Institute of Health, 10178 Berlin, Germany; 4 Engelhard Arzneimittel GmbH & Co. KG, 61138 Niederdorfelden, Germany; 5 Epidemiology, IQVIA, 60549 Frankfurt Am Main, Germany

**Keywords:** EA 575, Prospan, common cold, respiratory infection, antibiotics

## Abstract

Background: Dried ivy leaf extract EA 575^®^ (Prospan^®^) is commonly used to treat coughs and may help reduce inappropriate antibiotic use for the common cold. This retrospective study investigated whether prescribing EA 575 is associated with reduced subsequent antibiotic use in children and adolescents with the common cold. Repeated EA 575 prescriptions were also analyzed to estimate treatment satisfaction. Methods: Data were sourced from the IQVIA Disease Analyzer database, including patients under 18 diagnosed with a common cold and prescribed either EA 575 or antibiotics between 2017 and 2020 (index date). Propensity score matching controlled for confounding factors. Antibiotic prescriptions were assessed 4–30 and 31–365 days after the index date, along with bacterial infections 4–40 days post-index. Repeated EA 575 prescriptions 2–5 years post-index were analyzed as a proxy for treatment satisfaction. Results: Overall, 10,390 children and adolescents were included in each matched cohort. Compared to antibiotics, EA 575 prescriptions were associated with significantly lower odds of antibiotic use 4–30 days (OR: 0.56; 95% CI: 0.49–0.64; *p* < 0.001) and 31–365 days (OR: 0.58; 95% CI: 0.54–0.62; *p* < 0.001) after the index date. The odds of bacterial infection 4–30 days after EA 575 prescription were also lower (OR: 0.67; 95% CI: 0.45–0.99; *p* = 0.047). Of the 42,677 patients in the EA 575 analysis, 50.5% had at least one repeated prescription, with the highest rates among children aged 0–2 years (54.7%) and the lowest in those aged 13–17 years (19.9%). Conclusions: EA 575 prescription was associated with reduced subsequent antibiotic use in children and adolescents with common colds. Frequent repeated prescription rates emphasize the therapeutic value of EA 575 as a treatment option for cold symptoms, especially in younger children.

## 1. Introduction

The ‘common cold’ is an umbrella term for respiratory tract infections of the nose, throat, larynx, and bronchi [1]. A diverse range of symptoms are associated with the common cold, including cough, sore throat, and sneezing [2]. This variety can be partly attributed to the large number of viral strains that can cause a cold [1].

Common colds are highly prevalent in the pediatric population. According to findings from the German Health Interview and Examination Survey for Children (KiGGS), 88.5% of 17,641 children and adolescents had experienced at least one cold in the past year [3]. Moreover, the incidence of common colds tends to vary between age groups, with pre-school children experiencing up to 10 episodes per year, compared with 2–4 episodes per year in adolescents [4]. Since self-limiting viral infections are the predominant cause of common colds, treatment options are generally restricted to symptomatic interventions and antibiotics are ineffective [4,5,6]. Nevertheless, a retrospective cohort study found a high rate of antibiotic prescriptions in German pediatric practices, particularly during the winter months, suggesting frequent inappropriate use for the treatment of viral infections [7]. Inadequate and inappropriate use of antibiotics is known to contribute to bacterial resistance—a problem that poses an ever-increasing threat to mankind [8,9].

Symptomatic treatment of the common cold frequently involves self-medication with over-the-counter medicines [10]. Preparations of common ivy leaf (*Hedera helix*) dry extracts are well established in the treatment of respiratory disease [11]. Current guidelines from the German Respiratory Society cite evidence supporting the use of ivy leaf dry extracts for the treatment of cough in adults [12]. These guidelines also note that the therapeutic effects of phytotherapeutics, such as ivy leaf dry extracts, are specific to individual preparations and not interchangeable [12].

EA 575 is a preparation of dried ivy leaves (drug extract ratio 5–7.5:1) [13] that has been studied extensively in preclinical and clinical settings. Data suggest that EA 575 has bronchodilatatory [14,15,16] and secretolytic effects [14,17], and may also display anti-inflammatory properties [18,19,20]. These effects have been shown to translate into clinical benefits for the treatment of cough in adults and children [21,22]. In a prospective observational study performed in Latin America with 9657 patients (including 5181 children), EA 575 was considered generally effective and well tolerated for the treatment of bronchitis in routine clinical practice [23]. Furthermore, a prospective, observational study in 1066 school children demonstrated improvement of acute bronchitis after a 7-day treatment period with EA 575 [24], and a large retrospective study of 52,478 children supported an overall good tolerability profile for EA 575 [25].

The value of real-world studies for supporting healthcare decision-making is increasingly recognized across therapy areas [26]. While randomized controlled trials (RCTs) remain the ‘gold standard’ for assessing the efficacy and safety of an intervention, they lack external validity due to strict inclusion and exclusion criteria [27]. Conversely, real-world studies include a more heterogeneous patient population, allowing the evaluation of a treatment in routine clinical practice [27,28]. For payers and regulators, real-world data can fill evidence gaps that are not addressed by RCTs and support existing findings in particular areas of concern, such as long-term safety and effectiveness, and patient-reported outcomes [26]. Among other sources, healthcare databases are common repositories of real-world data for analysis [27].

A retrospective real-world analysis of the IMS^®^ Disease Analyzer database found that phytopharmaceutical use in adults and children with acute lower and upper respiratory tract infections was associated with a significantly reduced need for antibiotic prescription later in the course of disease [29]. Furthermore, a recent study based on real-world prescription data for adults with common cold diagnoses indicated that EA 575 prescription was associated with significantly lower odds of antibiotic prescription in the following year compared with matched patients who received an initial antibiotic [30]. Since most cases of bronchitis are caused by viruses [31], the findings from these studies lead to the hypothesis that there is a statistical relationship between the prescription of EA 575 and the subsequent prescription of antibiotics in patients with the common cold. However, there are currently no studies that evaluate this hypothesis specifically in children and adolescents—a population in which acute respiratory infections are common. Thus, the present, real-world study aims to determine whether or not such a relationship exists in the pediatric population. In addition, the study investigates the rate of repeated prescription of EA 575, prescribed for subsequent respiratory infections, according to the patient age group.

## 2. Materials and Methods

### 2.1. Hypothesis Generation

This retrospective matched cohort study was conducted to evaluate the hypothesis that there is a statistical relationship between the prescription of EA 575 and the subsequent prescription of antibiotics in pediatric patients with a common cold diagnosis. The hypothesis was generated a priori, based on the existing literature, and in consultation with experts in the fields of respiratory medicine and pharmacology to ensure that it was theoretically sound and practically relevant.

### 2.2. Data Source

This study utilized data from the IQVIA Disease Analyzer (DA) database. The IQVIA DA database contains case-based information provided by office-based physicians (general practitioners, pediatricians, and specialists) in Germany, including patient demographics, diagnoses, prescriptions, and reported sick leave. The quality of the data is regularly assessed by IQVIA on a number of criteria, for example, the completeness of the documentation and the link between diagnoses and prescriptions. The panel of practices included in the DA database is representative of general and specialized practices in Germany [32].

### 2.3. Study Population

The study population comprised children and adolescents (aged <18 years) from 220 pediatric practices who had at least one common cold diagnosis during the index time period (1 January 2017 to 31 December 2020): acute upper respiratory tract infection (URTI) of multiple and unspecified sites (International Classification of Disease, tenth revision [ICD-10]: J06); acute bronchitis (ICD-10: J20); bronchitis, not specified as acute or chronic (ICD-10: J40); cough (ICD-10: R05); acute nasopharyngitis (ICD-10: J00); or viral infection of unspecified site (ICD-10: B34). Analysis inclusion criteria were 1) availability of patient data for ≥12 months prior to the index date and 2) prescription of either EA 575^®^ (licensed under the trade name Prospan^®^, in Germany) (EA 575 cohort) or any systemic antibiotic drug (ATC: J01) (antibiotic cohort) on or ≤3 days after the index date (date of diagnosis). To account for confounding variables, patients with an antibiotic prescription or a diagnosis of a bacterial infection ≤ 30 days prior to the index date, and patients with a prescription of other herbal medicines (most commonly, Bronchipret^®^ and Bronchicum^®^), acetylcysteine, or ambroxol for the treatment of cough on or ≤3 days after the index date were excluded from the analyses. For the analysis of repeated prescriptions of EA 575, patients with at least one common cold diagnosis (as defined above) and a prescription of EA 575 between 1 January 2016 and 31 December 2018 and availability of data for ≥730 days after the first prescription were included.

### 2.4. Outcomes

Outcomes were the proportion of patients with at least one new antibiotic prescription 4–30 days and 31–365 days after the index date, and the proportion of patients with a diagnosed bacterial infection 4–30 days after the index date, for the EA 575 cohort versus the antibiotic cohort. Bacterial infection diagnoses comprised pneumococcal/staphylococcal pharyngitis (ICD-10: J02.0); streptococcal tonsilitis (ICD-10: J03.0); acute tonsilitis due to other specified organisms (ICD-10: J03.8); pneumonia due to streptococcus pneumoniae (ICD-10: J13); pneumonia due to hemophilus influenzae (ICD-10: J14); bacterial pneumonia, not elsewhere classified (ICD-10: J15); acute streptococcal bronchitis (ICD-10: J20.2); streptococcus and staphylococcus as the cause of diseases classified to other chapters (ICD-10: B95); and other specified bacterial agents as the cause of diseases classified to other chapters (ICD-10: B96).

In addition, in the EA 575 cohort, the number of repeated prescriptions of EA 575 for new infections in the 2–5-year follow-up period after the index date were quantified from patient records and were stratified by age group. Repeated prescriptions were used as a proxy to investigate previous positive experiences of EA 575 for patients or caregivers and physicians.

### 2.5. Propensity Score Matching and Statistical Analyses

To control for confounding, 1:1 matching without replacement was conducted based on a propensity score that is constructed as the conditional probability of being prescribed EA 575 as a function of age, sex, index month, diagnosis, and treating practice (logistic regression). Greedy matching was used, by which patients in the EA 575 cohort were matched with patients in the antibiotic cohort with the closest propensity score. Baseline demographics and clinical characteristics of the matched cohorts were presented using descriptive statistics, and group differences (EA 575 versus antibiotic) were analyzed using a Wilcoxon test (for continuous variables) or a Chi^2^ test (for categorical variables). A univariable logistic regression model was used to investigate the association between EA 575 prescription and new antibiotic prescription stratified by patient age group (0–2 years, 3–5 years, 6–12 years, and 13–17 years) and sex. The same model was used to investigate the association between EA 575 prescription and subsequent diagnosis of bacterial infection. Findings from the model were presented as odds ratios (ORs) with 95% confidence intervals (CI). A *p*-value of <0.05 was considered statistically significant. Analyses were conducted using SAS software version 9.4 (SAS Institute, Cary, NC, USA).

## 3. Results

### 3.1. Baseline Characteristics of Study Population

**Figure 1:**
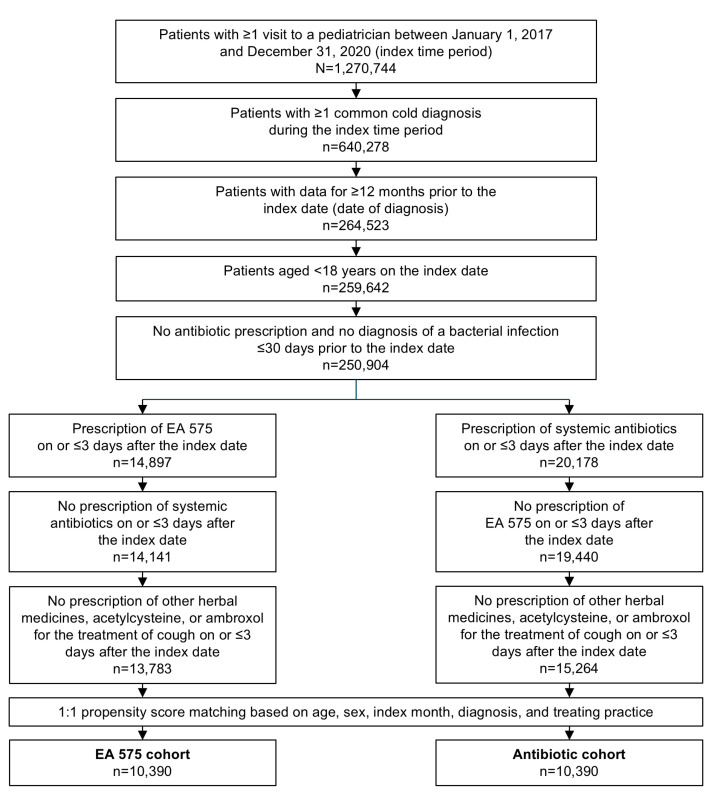
Selection of the study population.

Selection of the study population is shown in Figure 1. Of the 259,642 patients aged <18 years with a common cold diagnosis, 5.7% (14,897) received a prescription of EA 575. Before matching, 26.3% of patients in the antibiotic cohort were diagnosed with acute bronchitis versus 8.5% of patients in the EA 575 cohort. Following patient selection and propensity score matching, a total of 10,390 patients in the EA 575 cohort and 10,390 patients in the antibiotic cohort were identified for subsequent analysis (Figure 1).

The baseline characteristics of the matched cohorts are summarized in Table 1. Following pair-matching, there were no significant differences between the EA 575 and antibiotic cohorts in mean age (5.7 years versus 5.8 years), sex (both 48.7% female), or in the distribution of diagnoses (both 54.3% acute URTIs).

### 3.2. New Antibiotic Prescriptions

At least one new antibiotic prescription was issued 4–30 days after the index date for 3.7% of patients with a prior EA 575 prescription and for 6.5% of patients with a prior antibiotic prescription (Table 2). Within 31–365 days after the index date, at least one antibiotic prescription was issued for 19.4% of patients with a prior EA 575 prescription and 29.3% of patients with a prior antibiotic prescription (Table 2). Thus, a prior EA 575 prescription was associated with lower odds of a subsequent antibiotic prescription 4–30 days after the index date (OR: 0.56; 95% CI: 0.49–0.64; *p* < 0.001) and 31–365 days after the index date (OR: 0.58; 95% CI: 0.54–0.62; *p* < 0.001). This association was found across all investigated age groups, with the exception of patients aged 13–17 years in the 4–30-day time period (OR: 0.91; 95% CI: 0.40–2.10; *p* = 0.832).

### 3.3. Documented Bacterial Infections

Diagnoses of bacterial infections were generally rare in both cohorts. A bacterial infection was documented 4–30 days after the index date in 40 (0.38%) patients in the EA 575 cohort and in 60 (0.58%) patients in the antibiotic cohort. Although the absolute numbers were small, a prior EA 575 prescription was associated with lower odds of a bacterial infection 4–30 days after the index date (OR: 0.67; 95% CI: 0.45–0.99; *p* = 0.047).

### 3.4. Repeated EA 575 Prescriptions

**Figure 2:**
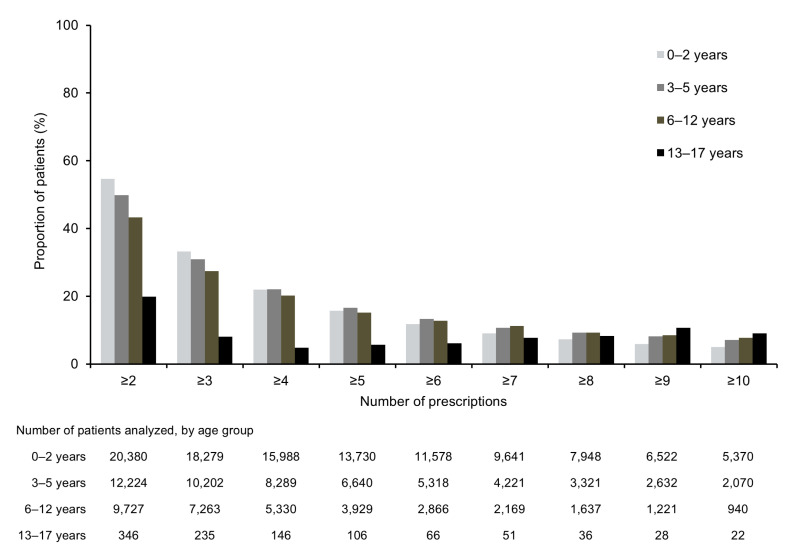
Documented instances of repeated prescriptions of EA 575 in the 2–5-year follow-up period, stratified by age group. The Y-axis shows the proportion of children with repeated prescriptions. The total 100% represents all children who received at least one EA 575 prescription.

A total of 42,677 patients were included in the EA 575 repeated prescription analysis. In the 2–5 years after the index date, 21,552 (50.5%) children and adolescents received at least one repeated prescription of EA 575 for a new infection. At least two repeated prescriptions were reported for 31.3% (n = 13,358) of children and adolescents. The rate of repeated prescriptions of EA 575 differed according to patient age (Figure 2). While 19.9% of patients aged 13–17 years had at least one repeated prescription, the corresponding value in patients aged 0–2 years was 54.7% (Figure 2).

## 4. Discussion

Based on this analysis of retrospective prescription data from over 20,000 children and adolescents, the use of EA 575 versus antibiotics for the treatment of the common cold was associated with significantly lower odds of subsequent antibiotic use and new bacterial infections. Additionally, more than 50% of children and adolescents with a prescription of EA 575 had at least one repeated prescription within the 2–5-year follow-up period and repeated prescription rates were particularly high in younger children (aged 0–2 years). Together, these data show possible benefits for EA 575 versus inappropriate use of antibiotics for the common cold and suggest a positive view of the therapeutic value of EA 575 by patients, their parents or caregivers, and by physicians.

Previous studies have provided evidence for the efficacy and safety of EA 575 in children compared with active controls such as acetylcysteine and ambroxol (summarized in Seifert et al., 2023) [22]. Additionally, in an observational study reported by Fazio et al., it was found that concomitant treatment with antibiotics increased the relative risk of adverse effects in children treated with EA 575 [23]. In the present dataset, while diagnoses of subsequent bacterial infections were rare, a higher proportion of patients in the antibiotic cohort experienced subsequent bacterial infections than in the EA 575 cohort. This observation might be the result of differences in the treating physicians’ clinical impression of bacterial infection in the antibiotic cohort versus the EA 575 cohort. However, the finding could be attributed to an increased susceptibility to infection in the antibiotic cohort, either due to external influencing factors (e.g., physical or socioeconomic environment), or a compromised immune system as a direct consequence of antibiotic use [33]. It could also be hypothesized that the bronchodilatatory [14,15,16] and secretolytic [16,17] effects of EA 575 have a role in the prevention of secondary bacterial infection, through improved airway clearance.

The present analysis showed an association between prior EA 575 prescription and reduced antibiotic prescriptions among children and adolescents in the follow-up period. This finding is in-line with the results from a database analysis for adult patients who received EA 575 for the treatment of a common cold [30]. Furthermore, a retrospective analysis conducted by Martin et al. suggested a reduced need for antibiotic prescription later in the course of acute respiratory infection among patients who had received phytopharmaceuticals (including ivy leaf dry extract preparations) [29]. While the severity of the diagnoses reported in the present analysis are not documented in the DA database, it may be hypothesized that physicians tend to prescribe antibiotics to patients with more severe disease. However, a case–control study has shown that the practice preference of the physician is one of the most significant factors influencing the decision to prescribe antibiotics for acute respiratory tract infections [34].

In pediatric practices, specifically, the variables showing the strongest association with the prescription of antibiotics were diagnoses of acute sinusitis or bronchitis [34]. In the present study, before pair-matching, 26.3% of patients in the antibiotic cohort received a diagnosis of acute bronchitis, compared with only 8.5% of patients in the EA 575 cohort. It is important to note that, although frequently prescribed in cases of acute bronchitis [35], antibiotics are ineffective for the treatment of viral infections [6], and the practice of antibiotic overprescription may contribute to antibiotic resistance [8]. Considering that acute bronchitis is most frequently caused by viral infections, antibiotic treatment would be expected to have minimal benefit in such cases, and could have harmful side effects [36].

In the present analysis, of the 259,642 patients aged <18 years with a common cold diagnosis, 5.7% (14,897) received an EA 575 prescription. Similarly, a German, survey-based study reported a 5.8% prevalence rate for all herbal medicine use among children and adolescents [37]. More than two-thirds of these cases (71.9%) involved treatment for coughs and colds and, in line with our data, a higher prevalence of herbal medicine use was observed among younger children [37]. Du et al. suggested that the high usage of herbal medicine in younger children (aged < 6 years) could reflect the broad acceptance of such medicines on account of their tolerability profile [37]. The safety and tolerability of a medication is of central importance in pediatric populations, especially when used for conditions that are typically self-limiting, such as the common cold. In the context of self-limiting illnesses, waiting for the illness to resolve without intervention would be an option, thus affecting the risk–benefit assessment of potential pharmacotherapies. Consequently, it is vital that the safety and tolerability of medicines for self-limiting conditions is investigated. For EA 575, safety and tolerability has been well documented in the literature, particularly in pediatric patients [22].

In the present study, analysis of repeated prescriptions provides some insights into the perceived therapeutic value of EA 575 among children, caregivers, and physicians in Germany. Overall, 50.5% (n = 21,552) of children and adolescents received at least one repeated prescription of EA 575 and 31.3% (n = 13,358) received at least two repeated prescriptions, suggesting a positive view of its therapeutic value (efficacy and tolerability) by caregivers and prescribers. This is supported by data from a retrospective review of >52,000 pediatric patient files, in which the reported incidence of adverse drug reactions was 0.2% [25]. A narrative review of pediatric trials and observational studies also reported that a high proportion of patients (≥95%) and clinicians (≥96%) rated the tolerability of EA 575 as good or very good [22]. When stratified by age group, the proportion of children with at least one repeated prescription of EA 575 was higher among children aged 0–2 years (54.7%) than among older children and adolescents aged 13–17 years (19.9%). This observation is a likely consequence of the known epidemiology of the common cold, which shows a higher annual incidence in younger versus older children [4]. Although overall rates of repeated EA 575 prescription were relatively high in the present analysis, it is important to highlight that this is likely an underestimate of actual medicine reuse. In Germany, EA 575 is available as an over-the-counter medicine, thus, patients can access the medicine without needing a prescription. In addition, the dataset is not able to capture the total number of repeated prescription events for patients who have changed their pediatrician or have shifted into another age cohort during the follow-up period. Therefore, the reported repeated prescription rates represent conservative estimates that are likely lower than the actual rates.

Key strengths of this study are the high number of patients and practices included, and the employment of real-world data that is able to capture the use of EA 575 in routine clinical practice, with a heterogenous patient group, over several years. However, the present findings must be interpreted with caution given the limitations of the study. First, assessments are based on ICD-10 codes entered by physicians, and some diagnostic codes do not allow for the separation of viral and bacterial etiologies (for example, acute bronchitis [ICD-10: J20]). Although acute bronchitis is usually diagnosed clinically based on medical history and physical examination alone, without additional diagnostic tests, most cases are attributed to viral infections [31,36]. Second, information on the severity of the disease was unknown and a general association between disease severity and antibiotic prescriptions cannot be ruled out. Similarly, the analysis was unable to capture additional physician- and practice-level characteristics that may have influenced the decision to prescribe antibiotics. Indeed, research has suggested that in children, time-related pressures and concerns about the potential for severe disease may encourage inappropriate antibiotic prescription by pediatricians [38]. Third, information on concomitant self-medication with non-prescription drugs is missing. Fourth, data on socioeconomic status and lifestyle-related risk factors (like physical activity and exposure to tobacco smoke) were not available. Finally, information about any additional diagnoses or prescriptions from doctors other than those from whom the patient records originate is missing.

In summary, the present study highlights the potential role of EA 575 in the reduction in subsequent antibiotic use in children and adolescents with the common cold. Furthermore, high rates of EA 575 repeated prescription indicate that EA 575 is a valuable treatment option for cold symptoms such as cough, especially in younger children.

## Figures and Tables

**Table 1:** Baseline demographics and clinical characteristics of the matched study cohorts.

Variable	EA 575 Cohort	Antibiotic Cohort	*p*-Value
n	10,390	10,390	–
Mean (SD) age, years	5.7 (3.4)	5.8 (3.5)	0.570
0–2 years, n (%)	2006 (19.3)	2006 (19.3)	1.000
3–5 years, n (%)	3670 (35.3)	3670 (35.3)	
6–12 years, n (%)	4380 (42.2)	4380 (42.2)	
13–17 years, n (%)	334 (3.2)	334 (3.2)	
Female, n (%)	5060 (48.7)	5060 (48.7)	1.000
Diagnosis			
Acute URTIs of multiple and unspecified sites (ICD-10: J06), n (%)	5643 (54.3)	5643 (54.3)	1.000
Acute bronchitis (ICD-10: J20), n (%)	999 (9.6)	999 (9.6)	1.000
Bronchitis, not specified as acute or chronic (ICD-10: J40), n (%)	483 (4.6)	483 (4.6)	1.000
Other diagnoses including acute rhinopharyngitis, cough, and viral infection of unspecified site, n (%)	3265 (31.4)	3265 (31.4)	1.000

ICD, International classification of disease; SD, standard deviation; URTI, upper respiratory tract infection.

**Table 2:** Association between EA 575 prescription and the probability of antibiotic prescription after the index date (EA 575 versus antibiotic).

Patient Subgroup	EA 575 Cohort, n (%)	Antibiotic Cohort, n (%)	OR (95% CI)	*p*-Value
n				
All patients	10,390	10,390	–	–
Female	5060	5060	–	–
Age 0–2 years	2006	2006	–	–
Age 3–5 years	3670	3670	–	–
Age 6–12 years	4380	4380	–	–
Age 13–17 years	334	334	–	–
New antibiotic prescription 4–30 days after the index date				
All patients	388 (3.7)	674 (6.5)	0.56 (0.49–0.64)	<0.001
Sex				
Female	183 (3.6)	321 (6.3)	0.55 (0.46–0.67)	<0.001
Male	205 (3.8)	353 (6.6)	0.56 (0.47–0.67)	<0.001
Age				
0–2 years	103 (5.1)	151 (7.5)	0.67 (0.51–0.86)	0.002
3–5 years	167 (4.6)	274 (7.5)	0.59 (0.49–0.72)	<0.001
6–12 years	107 (2.4)	237 (5.4)	0.44 (0.35–0.55)	<0.001
13–17 years	11 (3.3)	12 (3.6)	0.91 (0.40–2.10)	0.832
New antibiotic prescription 31–365 days after the index date				
All patients	2015 (19.4)	3045 (29.3)	0.58 (0.54–0.62)	<0.001
Sex				
Female	985 (19.5)	1484 (29.3)	0.58 (0.53–0.64)	<0.001
Male	1030 (19.3)	1561 (29.3)	0.58 (0.53–0.63)	<0.001
Age				
0–2 years	461 (23.0)	718 (35.8)	0.54 (0.47–0.62)	<0.001
3–5 years	885 (24.1)	1214 (33.1)	0.64 (0.58–0.71)	<0.001
6–12 years	637 (14.5)	1033 (23.6)	0.55 (0.49–0.62)	<0.001
13–17 years	32 (9.6)	80 (24.0)	0.34 (0.22–0.52)	<0.001

CI, confidence interval; OR, odds ratio.

## Data Availability

The data are not publicly available due to privacy restrictions.
